# University of Pennsylvania

**Published:** 1843-01-21

**Authors:** 


### CLINICAL LECTURES AND REPORTS.

### UNIVERSITY OF PENNSYLVANIA.

### CLINIC OF PROFESSOR HORNER.

*At the Locust St. Dispensary, Saturday, Jan. 7, 1843.*

#### LUXATION OF CERVICAL VERTEBRÆ.

Prof. Horner presented to the Class an example of recent occurrence of this rare and commonly fatal accident. A boy, Thomas Brierly, aged ten years, in clambering about an unfinished house, towards the latter part of November, missed his footing on the second floor and fell through the stairway to the cellar, head foremost, a distance of twenty feet. He was stunned by the fall, and was found, as stated by the friends, with his head bent under his body. He was conveyed senseless and motionless to his home. He gradually regained his perceptions but in an incoherent and perplexed manner: his head was much bruised by the fall, his neck was stiff and distorted, forming a large serpentine bulge on the left side, and a deep concavity to the right; his face was inclined downwards to the right side. Circumduction of the head was arrested and the neck motionless. The practitioner, Dr. Henry, who attended him in this stage of the injury, applied leeches to the part, emollients and frictions in succession.

In two days after the accident his common and accurate perceptions returned, but he was affected for some time with tingling and numbness in the left upper extremity.

At the period of his exhibition to the Class the deformity of the neck is still obvious, but much reduced; the rotatory motions of the neck can now be executed to some extent, but are much more to the right than to the left side. On tracing the line of the transverse processes of the vertebræ, the upper ones starting from the fourth are about half an inch forward of the lower, indicating clearly the advance of the vertebræ on that side, and consequently proving that the left lower oblique process of the fourth vertebra had been luxated in advance of the upper oblique of the fifth, and was there fixed.

We may presume from this state of the accident, that the intervertebral substance there had been partially or wholly ruptured, and that the two vertebræ were held together by the other ligamentous attachments and by the muscles. An attempt at the replacing of such a luxation is viewed with great apprehensions by surgeons. Dessault, in a case analogous, absolutely declined making the effort, for fear of its fatal issue; and it is related by M. Petit Radel, (Note Boyer Malad. Chir. vol. 4, p. 118,) that a young patient at La Charité expired in the hands of the surgeons, upon such an attempt a few days after the accident. This result is very intelligible when we reflect that to disengage an oblique process thus placed, it is necessary to begin by increasing the inflection forwards, or, in other words, by augmenting the displacement, which must in all probability tear up more of the natural fastenings of the bones, and thus subject the spinal marrow to compression or even laceration. Under these circumstances Brierly was dismissed with some general directions for his treatment, and the expectation that his youth would insure a still further erection of that part of the spine. At the period of his exhibition, say about six weeks after the accident, his general health was good, he enjoyed the use of all his faculties, and was going to school.

